# Guillain-Barre syndrome associated with previous dengue fever: case report

**DOI:** 10.17843/rpmesp.2025.421.13893

**Published:** 2025-03-17

**Authors:** Manuel Sanchez-Landers, Arnold Frank Rodriguez-Benites

**Affiliations:** 1 Faculty of Medicine, National University of Trujillo, Trujillo, Peru. National University of Trujillo Faculty of Medicine National University of Trujillo Trujillo Peru; 2 Emergency and Critical Care Department of Belén Hospital in Trujillo. Trujillo, Peru. Emergency and Critical Care Department of Belén Hospital in Trujillo Trujillo Peru

**Keywords:** Polyneuropathy, Arbovirus Infection, Arthropod Vectors, Intravenous Immunoglobulins, Peru

## Abstract

Guillain-Barré syndrome is a potentially severe autoimmune inflammatory polyneuropathy, which is usually associated with previous infections. Dengue is the most common arbovirus infection worldwide, being endemic in tropical and subtropical areas such as Peru. Scientific literature shows some reports of dengue with neurological complications, but its pathophysiology is not well understood. We present the case of a woman from an area endemic for dengue with Guillain-Barré syndrome with a disability scale of 4/6 on the Hughes scale, confirmed by albumin-cytological dissociation, and previous infection with dengue virus confirmed with a positive serum ELISA IgM test. The patient’s progress was favorable after receiving intravenous immunoglobulin. It is important to consider this viral infection as a probable risk factor for developing Guillain-Barré syndrome.

## INTRODUCTION

Dengue virus (DENV) is an arbovirus that is mostly transmitted by female *Aedes aegypti* arthropods. There are four serotypes, with serotypes DENV-1, DENV-2 and DENV-3 being the most frequent in Peru. The most common clinical manifestations are fever, joint pain, muscle pain, headache, skin reddening and morbilliform eruptions ^1^.

The spectrum of clinical manifestations related to dengue has transformed in recent years, especially regarding serotypes DENV-2 and DENV-3, with a higher frequency of neurological complications, encephalopathy or encephalitis being the most common, between 0.5 and 6.2%. Although the virus was initially considered to be non-neurotropic, neurological affection has been demonstrated by the detection of viral antigen in the brain through immunohistochemistry in fatal cases of dengue encephalopathy, as well as by polymerase chain reaction (PCR) and IgM antibody tests in the cerebrospinal fluid of patients with dengue encephalitis ^2^.

On the other hand, Guillain-Barré syndrome (GBS) is an acute inflammatory polyneuropathy that can occur after certain infections, such as gastrointestinal or upper respiratory infections. Although rare, there have been reports of GBS related to previous dengue. However, the epidemiological evidence and the underlying pathophysiological mechanisms are not yet fully understood ^3^.

This report analyzes the case of a patient with a previous dengue infection with no alarm signals and the subsequent development of moderate GBS, the early identification of which allowed for the administration of specific and timely treatment and avoided admission to the intensive care unit and the need for mechanical ventilation.

## CASE REPORT

### Patient information

40-year-old housewife, of mixed race, with no previous comorbidities, from an area endemic for dengue; who, 14 days before, presented temperature of 39°C, general malaise, headache, myalgia, arthralgia and retroocular pain; which improved with paracetamol intake and lasted four days.

Three days after the fever subsided, she gradually developed tingling paresthesia in both hands, with numbness and mild weakness in both upper limbs symmetrically. The next day, mild weakness in both lower limbs appeared, which did not limit daily activities. However, as the days passed, the weakness increased, predominantly in the lower limbs, with difficulty walking and then inability to stand or walk. She did not have any problems with bladder or bowel control at any time during the illness.

Due to prostration, the patient was admitted in a wheelchair to the emergency department of our hospital, hemodynamically stable and without respiratory distress. She denied chronic diseases, exposure to heavy metals, or recent immunizations.

### Clinical findings

The neurological examination showed that the patient was lucid, with symmetrical facial features, in a seated position due to inability to stand or walk, with quadriparesis predominantly in the lower limbs (MRC (Medical Research Council) strength scale: upper limbs 4/5; lower limbs 3/5), generalized flaccidity, bilateral biceps and triceps hyporeflexia, bilateral patellar and Achilles reflexes, no Babinski sign or sensory level, absence of meningeal signs, and normal segmental coordination. Pupils were isochoric and photoreactive, with no palpebral ptosis, normal eye movements, corneal and nauseous reflexes were present, and no dysarthria or dysphagia (Table 1).

**Table 1:** Clinical progression after receiving intravenous human immunoglobulin.

Clinical characteristics	One day before starting immunoglobulin	Twelve days after starting immunoglobulin
Upper limb weakness (MRC scale)	4/5	5/5
Lower limb weakness (MRC scale)	3/5	4/5
Deep reflexes of upper limbs	Hyporeflexia	Normal reflexes
Deep reflexes of lower limbs	Areflexia	Hyporeflexia
Facies	Symmetrical	Symmetrical
Eye movements	Normal	Normal
Autonomic disorders	Absent	Absent
Breathing difficulty	Absent	Absent
Standing and Walking	Prostrate in a wheelchair	Walk with a walker

MRC scale: Medical Research Council strength scale.

### Diagnostic evaluation and therapeutic intervention

The complete blood count, which included platelet count, glucose, urea, creatinine, and serum electrolytes, including potassium, liver function tests, and total creatine kinase, were normal. Therefore, no further tests were requested. Given the picture consistent with GBS, with a diagnostic certainty level of 3 according to the Brighton criteria and a Hughes disability scale of 4/6, urgent treatment started with 5% human immunoglobulin intravenously at a dose of 0.4 mg/kg. The patient was then admitted to the neurology department.

### Follow-up and results

During her hospital stay, the patient completed five days of treatment with intravenous immunoglobulin and began physical therapy. After seven days of hospitalization, a lumbar puncture showed cerebrospinal fluid with 2 cells per mm^3^ and cerebrospinal fluid protein level of 178 mg/dL, confirming the diagnosis of GBS. Electromyography and nerve conduction studies were not performed due to lack of equipment at our hospital. Given the history of self-limiting fever prior to the neurological symptoms and the fact that the patient came from an area endemic for dengue, a serum ELISA test for dengue was performed, with a positive IgM result. No other etiology for GBS was sought due to lack of supplies. The patient did not present respiratory distress and was discharged after 12 days of hospitalization, with diminishing weakness, achieving bipedal stance and walking with a walker. A control lumbar puncture was not performed due to lack of patient authorization.

## DISCUSSION

GBS is the most common acute autoimmune inflammatory polyneuropathy worldwide, with a global prevalence of 1.9 cases per 100,000 inhabitants; it is more prevalent in developed countries in the Asia-Pacific region and North America, with rates of up to 6.4 and 4.2 cases per 100,000 inhabitants, respectively. The average rate is 2.5 cases per 100,000 inhabitants in South American countries. Severe disability due to GBS can occur in up to 20% of cases, and the mortality rate is 5% ^4^.

GBS is clinically characterized by progressive and symmetrical weakness of the limbs, generally ascending and of varying intensity, accompanied by generalized flaccidity and areflexia or hyporeflexia, over a period of 28 days. In addition, it may present with autonomic dysfunction or respiratory failure in the first seven days, which are serious and potentially fatal complications of this disease ^5^. The peculiarity of this case is its atypical presentation, as the weakness was descending, although predominantly in the lower limbs, with rapid progression to prostration at the end of the first week of illness, but without respiratory distress, which could have raised doubts about the initial diagnosis.

GBS has no known cause, but there are situations that precede the development of the condition, such as certain gastrointestinal or upper respiratory infections, which can trigger the onset of this disease in up to 66% of cases. The pathogen most frequently associated with GBS is *Campylobacter jejuni*, identified in approximately 30% of patients in Western countries and in about 50% in Asian countries. Other causes include infections by *Mycoplasma pneumoniae*, *Haemophilus influenzae*, influenza virus, hepatitis virus, cytomegalovirus, and Epstein-Barr virus ^6^. Our patient denied history of diarrhea or flu-like symptoms, although she did present an acute nonspecific fever syndrome a few days before the onset of neurological symptoms.

The theory of molecular mimicry is the most widely accepted explanation for the pathophysiology of this neurological disease following an infectious episode. Once the infection is controlled, the remaining circulating antimicrobial antibodies are directed, through a cross-reaction, against peripheral nerve structures, such as gangliosides, causing inflammatory damage to the myelin sheath or axon, giving rise to the clinical picture ^7^.

In recent years, cases of the association between GBS and certain viruses have been reported, such as SARS-CoV-2, Zika, chikungunya, and dengue ^8^^,^^9^. Although the incidence of GBS following Zika virus infection is higher, dengue is the most common arbovirus infection worldwide, with an estimated number of cases thirty times higher than in previous decades. It is also estimated that millions of symptomatic and asymptomatic infections occur each year, with thousands of deaths worldwide ^10^.

Dengue virus can cause neurological symptoms and signs through three mechanisms: direct damage caused by the virus invading the nervous system; indirect damage to the nervous system caused by systemic metabolic or vascular disturbances produced by the virus; and immune-mediated damage following viral infection, which causes the overproduction of proinflammatory cytokines by anti-ganglioside antibodies, with damage to the peripheral nerves. The first two occur during the febrile or critical phase, while the third appears during the convalescent phase ^11^. Our patient had fever consistent with dengue that resolved spontaneously prior to the onset of GBS and also came from a dengue-endemic area, so her neurological symptoms are likely explained by the latter mechanism.

Dengue virus infection can be detected by the presence of IgM in a serum ELISA test ^12^. The diagnosis of GBS is clinical; however, it is confirmed by evidence of albumin-cytological dissociation in the cerebrospinal fluid, and neurophysiological studies allow differentiation between demyelinating and axonal variants of GBS ^5^.

Since GBS is a post-infectious syndrome, specific treatment consists of therapies that eliminate harmful antibodies from the body, plasmapheresis, or intravenous immunoglobulin, but it must be administered early to reduce the severity and duration of the disease ^7^.

Most patients with GBS and previous dengue infection have demyelinating variants and negative serum antiganglioside antibody titers, with favorable progression of weakness after receiving specific treatment ^13^^-^^16^; although there are isolated reports of axonal variants with positive anti-GM1 antibodies ^17^ or Miller-Fisher variants ^18^.

In our case, treatment with intravenous immunoglobulin started early due to the rapid onset of GBS-induced prostration, with the aim of halting disease progression and preventing respiratory failure. In addition, dengue was suspected due to the previous fever and the fact that the patient came from an endemic area. Both conditions were confirmed by auxiliary tests, and the patient progressed favorably with the treatment.

Previous studies describe several case series of GBS in which the simultaneous presence of more than one infectious agent has been identified as a possible trigger for the neurological symptoms in the same patient ^19^^,^^20^. It is therefore important to study several possible etiologies. The main limitation in this case was that the etiological study was not completed and neurophysiological tests were not performed due to a lack of supplies and equipment in our hospital.

In conclusion, this report highlights the importance of considering differential diagnoses in the evaluation and management of GBS, particularly in regions endemic for viral diseases such as dengue. The coexistence of GBS and dengue in our patient illustrates the clinical complexity and the need for a comprehensive approach to the diagnosis and treatment of these cases.

## References

[1] Salles TS, da Encarnação Sá-Guimarães T, de Alvarenga ESL, Guimarães-Ribeiro V, de Meneses MDF, de Castro-Salles PF (2018). History, epidemiology and diagnostics of dengue in the American and Brazilian contexts a review. Parasit Vectors.

[2] Trivedi S, Chakravarty A (2022). Neurological Complications of Dengue Fever. Curr Neurol Neurosci Rep.

[3] Sheikh KA (2020). Guillain-Barré Syndrome. Continuum (Minneap Minn).

[4] Bragazzi NL, Kolahi AA, Nejadghaderi SA, Lochner P, Brigo F, Naldi A (2021). Global, regional, and national burden of Guillain-Barré syndrome and its underlying causes from 1990 to 2019. J Neuroinflammation.

[5] Shahrizaila N, Lehmann HC, Kuwabara S (2021). Guillain-Barré syndrome. Lancet.

[6] Dutta D, Debnath M, Nagappa M, Das SK, Wahatule R, Sinha S (2021). Antecedent infections in Guillain-Barré syndrome patients from south India. J Peripher Nerv Syst.

[7] Jasti AK, Selmi C, Sarmiento-Monroy JC, Vega DA, Anaya JM, Gershwin ME (2016). Guillain-Barré syndrome causes, immunopathogenic mechanisms and treatment. Expert Rev Clin Immunol.

[8] Pimentel V, Luchsinger VW, Carvalho GL, Alcará AM, Esper NB, Marinowic D (2023). Guillain-Barré syndrome associated with COVID-19 A systematic review. Brain Behav Immun Health.

[9] Lima MES, Bachur TPR, Aragão GF (2019). Guillain-Barre syndrome and its correlation with dengue, Zika and chikungunya viruses infection based on a literature review of reported cases in Brazil. Acta Trop.

[10] Khan MB, Yang ZS, Lin CY, Hsu MC, Urbina AN, Assavalapsakul W (2023). Dengue overview An updated systemic review. J Infect Public Health.

[11] Prabhat N, Ray S, Chakravarty K, Kathuria H, Saravana S, Singh D (2020). Atypical neurological manifestations of dengue fever a case series and mini review. Postgrad Med J.

[12] Carod-Artal FJ (2019). Complicaciones neurologicas asociadas a la infeccion por el virus del dengue. Rev Neurol.

[13] Tan CY, Razali SNO, Goh KJ, Sam IC, Shahrizaila N (2019). Association of dengue infection and Guillain-Barré syndrome in Malaysia. J Neurol Neurosurg Psychiatry.

[14] Lim CS, Kaisbain N, Lim WJ (2023). A Rare Combination Dengue Fever Complicated With Guillain-Barre Syndrome. Cureus.

[15] Dalugama C, Shelton J, Ekanayake M, Gawarammana IB (2018). Dengue fever complicated with Guillain-Barré syndrome a case report and review of the literature. J Med Case Rep.

[16] Simon O, Billot S, Guyon D, Daures M, Descloux E, Gourinat AC (2016). Early Guillain-Barré Syndrome associated with acute dengue fever. J Clin Virol.

[17] Prado MB, Narito KM, Adiao KJB (2021). Anti-GM1 IgM antibody positive axonal variant of Guillain-Barre-syndrome in a pediatric patient with dengue fever. J Neuroimmunol.

[18] de Silva NL, Weeratunga P, Umapathi T, Malavige N, Chang T (2019). Miller Fisher syndrome developing as a parainfectious manifestation of dengue fever a case report and review of the literature. J Med Case Rep.

[19] Hao Y, Wang W, Jacobs BC, Qiao B, Chen M, Liu D (2019). Antecedent infections in Guillain-Barré syndrome a single-center, prospective study. Ann Clin Transl Neurol.

[20] Pampa-Espinoza L, Mendoza N, Espinoza L, Flores-León D, Quino W, Gavilán R (2023). Características clínicas y laboratoriales de los diez primeros pacientes con diagnóstico de Guillain-Barré en Piura, 2023. An Fac med.

