# Supplementary material for: Guillain-Barre syndrome associated with previous dengue fever: case report
Source: Rev Peru Med Exp Salud Publica. 2025 Mar 17;42(1):88–91. doi: 10.17843/rpmesp.2025.421.13893 (PMC12176019; doi:10.17843/rpmesp.2025.421.13893)
Supplement: Translation

# Translation: Síndrome de Guillain-Barre asociado a dengue previo: reporte de caso

**Authors:** Manuel Sanchez-Landers, Arnold Frank Rodriguez-Benites

**Affiliations:** 1 Facultad de Medicina, Universidad Nacional de Trujillo, Trujillo, Perú.; 2 Departamento de Emergencia y Cuidados Críticos del Hospital Belén de Trujillo. Trujillo, Perú.

**Keywords:** Polineuropatía, infección por Arbovirus, Vectores Artrópodos, Inmunoglobulina Intravenosa, Perú

## Abstract

El síndrome de Guillain-Barré es una polineuropatía inflamatoria autoinmune potencialmente grave, que suele asociarse a infecciones previas. El dengue es la infección por arbovirus más frecuente a nivel mundial, siendo endémica en zonas tropicales y subtropicales como Perú. En la literatura científica existen algunos reportes de dengue con complicaciones neurológicas, pero su fisiopatología no está bien esclarecida. Presentamos el caso de una mujer procedente de una zona endémica de dengue con síndrome de Guillain-Barré con escala de discapacidad de Hughes 4/6, confirmado por disociación albúmino-citológica, e infección previa por virus del dengue confirmado con prueba sérica ELISA IgM positiva. La paciente presentó evolución favorable tras recibir inmunoglobulina intravenosa. Se destaca la importancia de considerar esta infección viral como probable factor de riesgo para desarrollar síndrome de Guillain-Barré.

## INTRODUCCIÓN

El virus del Dengue (DENV) es un arbovirus que se transmite predominantemente por artrópodos hembras de *Aedes aegypti*. Existen cuatro serotipos, siendo predominantes en Perú los serotipos DENV-1, DENV-2 y DENV-3. Las manifestaciones clínicas más frecuentes son fiebre, dolor articular, dolor muscular, dolor de cabeza, enrojecimiento de la piel y erupciones morbiliformes ^1^.

En los últimos años, se ha observado una transformación en el espectro de manifestaciones clínicas relacionadas con el virus del dengue, sobre todo en los serotipos DENV-2 y DENV-3, con mayor frecuencia de complicaciones neurológicas, siendo la encefalopatía o encefalitis las más comunes, entre 0,5 y 6,2%. Aunque inicialmente se consideró al virus como no neurotrópico, se ha demostrado neuroinvasión mediante la detección del antígeno viral en el cerebro a través de inmunohistoquímica en casos fatales de encefalopatía por dengue, así como mediante la reacción en cadena de la polimerasa (PCR) y pruebas de anticuerpos IgM en el líquido cefalorraquídeo de pacientes con encefalitis por dengue ^2^.

Por otro lado, el síndrome de Guillain-Barré (SGB) es una polineuropatía inflamatoria aguda que puede ocurrir después de ciertas infecciones, como las gastrointestinales o respiratorias altas. Aunque es poco frecuente, se han reportado casos de SGB relacionados con dengue previo. Sin embargo, la evidencia epidemiológica y los mecanismos fisiopatológicos subyacentes aún no están completamente claros ^3^.

En este reporte, se analiza el caso de una paciente con infección previa de dengue sin signos de alarma y desarrollo posterior de SGB moderado, cuya identificación temprana permitió administrar tratamiento específico oportuno y evitar su ingreso a la unidad de cuidados intensivos y la necesidad de ventilación mecánica.

## REPORTE DEL CASO

### Información del paciente

Mujer de 40 años, de raza mestiza y ocupación ama de casa, sin comorbilidades conocidas previas, procedente de zona endémica para dengue; quien hace 14 días presentó cuadro clínico de fiebre de 39 °C, malestar general, cefalea, mialgias, artralgias y dolor retroocular; que mejoró con ingesta de paracetamol y que duro cuatro días.

Tres días después de mejorar el cuadro febril, presentó de forma progresiva parestesias tipo hormigueo en las dos manos, con adormecimiento y debilidad leve en ambos miembros superiores de forma simétrica. Al día siguiente se agregó debilidad leve en ambos miembros inferiores que no limitaban las actividades cotidianas. Sin embargo, con el paso de los días la debilidad se incrementó, a predominio de miembros inferiores, con dificultad para caminar y luego con imposibilidad de bipedestación y marcha. No presentó problemas de control de esfínteres en ningún momento de la enfermedad.

Debido a la postración, la paciente ingresó en silla de ruedas al tópico de medicina del Servicio de Emergencia de nuestro hospital, hemodinámicamente estable y sin dificultad respiratoria. Negaba enfermedades crónicas, exposición a metales pesados o inmunizaciones recientes.

### Hallazgos clínicos

Al examen neurológico, la paciente se encontraba lúcida, con facie simétrica, en actitud de sedestación obligada por imposibilidad para bipedestación y marcha con cuadriparesia a predominio inferior (escala de fuerza MRC (Medical Research Council): miembros superiores 4/5; miembros inferiores 3/5), flacidez generalizada, hiporreflexia bicipital y tricipital bilateral, arreflexia rotuliana y aquiliana bilateral, sin signo de Babinski ni nivel sensitivo, ausencia de signos meníngeos y coordinación segmentaria normal. Pupilas isocóricas y fotorreactivas, sin ptosis palpebral, movimientos oculares normales, reflejos corneal y nauseosos presentes, sin disartria o disfagia (Tabla 1).

**Tabla 1:** Evolución clínica tras recibir inmunoglobulina humana intravenosa.

Características clínicas	Un día antes de empezar inmunoglobulina	Doce días después de empezar inmunoglobulina
Debilidad de miembros superiores (Escala MRC)	4/5	5/5
Debilidad de miembros inferiores (Escala MRC)	3/5	4/5
Reflejos profundos de miembros superiores	Hiporreflexia	Normorreflexia
Reflejos profundos de miembros inferiores	Arreflexia	Hiporreflexia
Facies	Simétrica	Simétrica
Movimientos oculares	Normales	Normales
Disautonomías	Ausentes	Ausentes
Dificultad respiratoria	Ausente	Ausente
Bipedestación y Marcha	Postrada en silla de ruedas	Camina con andador

Escala MRC: escala de fuerza Medical Research Council.

### Evaluación diagnóstica e intervención terapéutica

Se realizó un hemograma completo con recuento de plaquetas, glucosa, urea, creatinina y electrolitos séricos, incluyendo el dosaje de potasio, perfil hepático y creatinquinasa total, que resultaron normales. Por esta razón, no se solicitaron controles posteriores para estos exámenes. Por el cuadro compatible con SGB, con nivel de certeza diagnóstica de 3 según los criterios de Brighton y escala de discapacidad de Hughes de 4/6, se decidió iniciar tratamiento de forma urgente con inmunoglobulina humana al 5% por vía intravenosa a una dosis de 0,4 mg/kg. Posteriormente, la paciente fue hospitalizada en el servicio de neurología.

### Seguimiento y resultados

Durante su estancia hospitalaria, la paciente culminó los cinco días de tratamiento con inmunoglobulina intravenosa y empezó terapia física. Después de siete días de hospitalización, se le realizó punción lumbar, encontrándose líquido cefalorraquídeo con 2 células por mm^3^ y proteinorraquia de 178 mg/dL, lo que confirmó el diagnóstico de SGB. No se realizó electromiografía y conducción nerviosa por falta de equipo en nuestro hospital. Por el antecedente del cuadro febril autolimitado previo al cuadro neurológico y por ser procedente de una zona endémica para dengue, se efectuó prueba sérica de ELISA para dengue con resultado de IgM positivo. No se buscó otra etiología para SGB por falta de insumos. La paciente no presentó dificultad respiratoria y fue dada de alta después de 12 días de hospitalización, con evolución favorable de la debilidad, logrando bipedestación y marcha con andador. No se realizó punción lumbar de control por falta de autorización de la paciente.

## DISCUSIÓN

El SGB es la polineuropatía inflamatoria autoinmune aguda más frecuente a nivel mundial, con una prevalencia global de 1,9 casos por 100 000 habitantes; siendo más prevalente en países desarrollados de la región Asia-Pacífico y de Norteamérica donde alcanzan tasas de 6,4 y 4,2 casos por 100 000 habitantes, respectivamente. Mientras que, en países de Sudamérica, la tasa promedio es de 2,5 casos por 100 000 habitantes. La discapacidad severa por SGB puede ocurrir hasta en el 20% de los casos y la tasa de mortalidad es de 5% ^4^.

El SGB se caracteriza clínicamente por debilidad progresiva y simétrica de las extremidades, generalmente ascendente y de intensidad variable, acompañada de flacidez generalizada y arreflexia o hiporreflexia, durante un periodo de 28 días. Además, puede cursar con disautonomías o insuficiencia respiratoria en los primeros siete días, que son complicaciones graves y potencialmente mortales de esta enfermedad ^5^. La particularidad de este caso es su presentación atípica ya que la debilidad fue descendente, aunque a predominio de miembros inferiores, con rápida progresión a la postración al término de la primera semana de enfermedad, pero sin llegar a la dificultad respiratoria, lo que pudo generar dudas sobre el diagnóstico inicial.

El SGB no tiene una causa conocida, pero existen situaciones que preceden al desarrollo del cuadro, como algunas infecciones gastrointestinales o respiratorias altas que pueden desencadenar el inicio de esta enfermedad, hasta en el 66% de los casos. El agente patógeno más frecuentemente asociado con el SGB es *Campylobacter jejuni,* identificado en aproximadamente el 30 % de los pacientes en países occidentales y en cerca del 50 % en países asiáticos.. Otros agentes encontrados son *Mycoplasma pneumoniae*, *Haemophilus influenzae*, virus de Influenza, virus de hepatitis, citomegalovirus, virus de Epstein-Barr ^6^. Nuestra paciente negó antecedentes de cuadros diarreicos o gripales; aunque sí presentó un síndrome febril agudo inespecífico unos días antes del inicio de los síntomas neurológicos.

La teoría del mimetismo molecular es la más aceptada para explicar la fisiopatología de esta enfermedad neurológica tras un cuadro infeccioso. Una vez controlada la infección, los anticuerpos antimicrobianos circulantes remanentes se dirigen, mediante una reacción cruzada, contra estructuras de los nervios periféricos, como los gangliósidos, lo que provoca daño inflamatorio en la vaina de mielina o en el axón, dando lugar al cuadro clínico ^(^^7^.

En los últimos años, se ha demostrado una asociación entre el SGB y algunos virus, como el SARS-CoV-2, zika, chikungunya y dengue ^8^^,^^9^. Aunque la incidencia de SGB tras la infección por el virus Zika es mayor, el dengue es la infección por arbovirus más frecuente a nivel mundial, y se estima que el número de casos es treinta veces mayor en comparación con décadas pasadas. Asimismo, se calcula que cada año ocurren millones de infecciones sintomáticas y asintomáticas por este virus, con miles de muertes en todo el mundo ^10^.

El virus del dengue puede producir síntomas y signos neurológicos a través de tres mecanismos: daño directo por invasión del virus al sistema nervioso; daño indirecto del sistema nervioso por disturbios metabólicos o vasculares producidos por el virus de forma sistémica; daño inmunomediado posterior a la infección viral que ocasiona sobreproducción de citoquinas proinflamatorias por parte de los anticuerpos antigangliósidos, con lesión de los nervios periféricos. Los dos primeros ocurren en la fase febril o crítica; mientras que el tercero aparece en la fase de convalecencia ^11^. Nuestra paciente tuvo un cuadro febril compatible con dengue que se autolimitó previo al inicio del SGB y además era procedente de una zona endémica de dengue por lo que probablemente su cuadro neurológico es explicado por este último mecanismo.

La infección por el virus del dengue se puede detectar por la presencia de IgM en la prueba sérica de ELISA ^12^. El diagnóstico de SGB es clínico; aunque se confirma al evidenciar la disociación albúmino-citológica en el líquido cefalorraquídeo y los estudios de neurofisiología permiten diferenciar las variantes desmielinizantes o axonales del SGB ^5^.

Al ser un síndrome posinfeccioso, el tratamiento específico del SGB consiste en terapias que eliminen los anticuerpos dañinos del organismo, plasmaféresis o inmunoglobulina intravenosa, pero debe ser administrado de forma precoz para lograr reducir la gravedad y la duración de la enfermedad ^7^.

La mayoría de los pacientes con SGB e infección previa de dengue tienen variantes desmielinizantes y dosaje sérico negativo de anticuerpos antigangliósidos, con evolución favorable de la debilidad tras recibir tratamiento específico ^13^^-^^16^; aunque existen reportes aislados de variante axonal con anticuerpos anti-GM1 positivos ^17^ o variante Miller-Fisher ^18^.

En nuestro caso, se inició tempranamente inmunoglobulina intravenosa debido a la rapidez con la que el SGB había provocado la postración, con el objetivo de detener la progresión de la enfermedad y prevenir una insuficiencia respiratoria. Además, se sospechó dengue debido al cuadro febril previo y al hecho de que la paciente provenía de una zona endémica. Ambas patologías fueron confirmadas mediante exámenes auxiliares, y la paciente evolucionó favorablemente con el tratamiento instaurado.

En la literatura se han descrito algunas series de casos de SGB en las que se ha identificado la presencia simultánea de más de un agente infeccioso como posible desencadenante del cuadro neurológico en un mismo paciente ^19^^,^^20^. Por ello, es importante investigar diversas etiologías probables. La principal limitación en este caso fue que no se completó el estudio etiológico ni se realizaron pruebas neurofisiológicas debido a la falta de insumos y equipos en nuestro hospital.

En conclusión, este reporte destaca la importancia de considerar diagnósticos diferenciales en la evaluación y manejo del SGB, especialmente en regiones endémicas para enfermedades virales como el dengue. La coexistencia de SGB y dengue en nuestra paciente ilustra la complejidad clínica y la necesidad de un enfoque integral en el diagnóstico y tratamiento de estos casos.
